# Size Matters: Problems and Advantages Associated with Highly Miniaturized Sensors

**DOI:** 10.3390/s120303018

**Published:** 2012-03-06

**Authors:** Andreas B. Dahlin

**Affiliations:** Division of Bionanophotonics, Department of Applied Physics, Chalmers University of Technology, Fysikgränd 3, 41296 Göteborg, Sweden; E-Mail: adahlin@chalmers.se; Tel.: +46-3177-23228; Fax: +46-3177-22090

**Keywords:** sensor, biosensor, size, miniaturization, surface, nano, micro

## Abstract

There is no doubt that the recent advances in nanotechnology have made it possible to realize a great variety of new sensors with signal transduction mechanisms utilizing physical phenomena at the nanoscale. Some examples are conductivity measurements in nanowires, deflection of cantilevers and spectroscopy of plasmonic nanoparticles. The fact that these techniques are based on the special properties of nanostructural entities provides for extreme sensor miniaturization since a single structural unit often can be used as transducer. This review discusses the advantages and problems with such small sensors, with focus on biosensing applications and label-free real-time analysis of liquid samples. Many aspects of sensor design are considered, such as thermodynamic and diffusion aspects on binding kinetics as well as multiplexing and noise issues. Still, all issues discussed are generic in the sense that the conclusions apply to practically all types of surface sensitive techniques. As a counterweight to the current research trend, it is argued that in many real world applications, better performance is achieved if the active sensor is larger than that in typical nanosensors. Although there are certain specific sensing applications where nanoscale transducers are necessary, it is argued herein that this represents a relatively rare situation. Instead, it is suggested that sensing on the microscale often offers a good compromise between utilizing some possible advantages of miniaturization while avoiding the complications. This means that ensemble measurements on multiple nanoscale sensors are preferable instead of utilizing a single transducer entity.

## 1. Introduction

Recent advances in nanotechnology have made it possible to fabricate a great variety of structures with special optical, mechanical and electrical properties. These phenomena are characteristic of the nanoscale but can usually be explained fairly well by classical physics operating on homogenous materials. For instance, plasmon resonances in metallic nanostructures emerge as solutions to Maxwell’s equations [1,2], molecular adsorption can induce cantilever bending through surface stress [3] and high surface to volume ratios can cause the conductivity of semiconductors to depend on the external environment [4]. All these principles have been demonstrated to work as signal transduction mechanisms in surface-based analysis, leading to sensors operating through optical, mechanical or electrical detection [5]. It is important to note that the techniques are “blind” in the sense that they respond to accumulation of practically any molecule on the surface. This is because the signals are induced by more or less generic molecular properties such as refractive index, mass or charged groups. Therefore, sensor specificity relies heavily on efficient surface functionalization strategies [6]. Receptors must be immobilized and non-specific binding suppressed.

Most of the existing nanostructured sensors are based on or at least compatible with readout of a single structural entity. For instance, the scattering spectrum of a single plasmonic nanoparticle can be measured in the far field, the deflection of a single nanocantilever can be observed and the conductivity of a single nanowire can be probed electrically. This opens up for extreme sensor miniaturization, *i.e.*, *nanosensors* with an active detection area even below 1 μm^2^. There are plenty of reviews available that present various types of optical, mechanical and electrical surface sensitive techniques, including those compatible with miniaturization. However, the topic of sensor size itself is rarely discussed. Instead, miniaturization seems generally considered to be some sort of obvious advantage and a goal worth pursuing. There is rarely any questioning of *why* sensors should be as small as possible. Besides a few reports on theoretical predictions suggesting that nanosensors will suffer heavily from mass transport limitations [7,8], problems with miniaturization are rarely discussed. Instead, one can see somewhat of a “nanohype” in sensor research without any proper investigation of the pros and cons of highly miniaturized transducers.

This review aims to go through all the major advantages and disadvantages of having nanoscale sensors. I aim to describe how the *same* sensor would perform if it would have had a larger analyte capturing region. For many nanosensors, the sensitivity may be affected by altering the dimensions of the structural element. However, one can then ask the question whether measurements should be performed on more than one structural entity. This is essentially simply another way to change the effective size of the sensor and the sensitivity will not be affected (but the detection limit might). In other words, it is important to distinguish between finding the optimal geometry of an individual nanosensor and the number of such sensors that one should employ in the actual sensing application. The latter should in general not influence the signal transduction mechanism, but can provide noise reduction through averaging. I will limit myself to surface-based techniques that operate by adding some form of sample to the sensor, thereby excluding *in vivo* probes. In terms of exemplification, the discussion in this review is focused on detection of analytes in liquids and biosensor technologies, but the principles should hold also for chemical sensing in general as well as detection in gas phase. I focus on how miniaturization influences the performance of surface-based sensors in real applications. This means that I will consider what kind of samples and analytes one typically faces and what experimental data really look like when acquired from highly miniaturized transducers, usually a single nanosensor. Can the measurement precision be improved by measuring on more than one nanowire, nanocantilever or nanoparticle? If so, what are the implications in real sensing applications? I would say the discussion that follows is sometimes quite “down to earth” and not very complicated in a scientific sense. However, this does not make the conclusions any less important. In many cases, the text reflects over simple facts, but it seems many researchers do not to consider their implications. My hope is that a review of this kind can lead to a constructive debate over the use of highly miniaturized sensors.

## 2. Possible Advantages with Miniaturization

**Figure 1.**
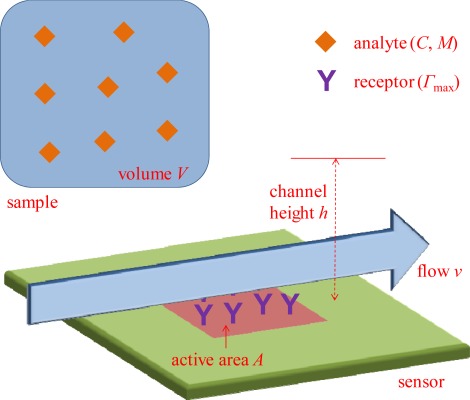
The principle of a surface-based sensor system operating *in vitro*. On the active area *A* there are recognition elements for the target analyte, which has molecular weight *M* and is present in a sample of volume *V* at concentration *C*. The sample solution is introduced to the surface in a channel with height *h*, possibly with continuous flow at an average velocity *v*. The maximum surface coverage of the analyte *Γ*_max_ is defined by the number of receptors (and *M* implicitly).

I will start by going through the common advantages of sensor miniaturization and evaluate to what extent they really enhance sensor performance (direct problems with miniaturization are discussed in the next section.). Consider the system in Figure 1. The subject of miniaturization concerns the sensor active area *A*, where analyte binding occurs and generates a signal. The sensor surface is exposed to a sample with volume *V*, in which we find the analyte present at a (molar) concentration *C*. The number of molecules available is then *VC* and the mass of analyte available is *VCM*, where *M* is the molecular mass. The sensor operates by transducing molecular adsorption on *A* into a detectable signal that increases (usually linearly) with the surface coverage *Γ* (mass per unit area). As a result of surface functionalization, there will be a certain number of binding sites available on *A* and there is a defined maximum surface coverage *Γ*_max_. Increasing *Γ*_max_ is one strategy to improve the performance of any surface-based sensor, but surface chemistry is not directly related to the miniaturization issue and will thus not be discussed further. Instead, I will here consider the consequences of the chosen size of *A*, especially in relation to *V*, *C* and *M*. For one thing I will show that the research field would likely benefit much from thinking more in terms of concentration instead of number of molecules. As will be shown, the quantity that determines *Γ* is normally *C*, while *V* does not come into play even for relatively large *A*.

Notably, the geometry of the sensor in Figure 1 is simplified in order to illustrate important points in a clear manner. Many sensors (e.g., nanowires) obviously do not have a simple planar rectangular detection region. However, the purpose here is to investigate the basic influence from *A* and derive scaling laws in relation to other parameters. For instance, a circular sensing region will only introduce another proportionality factor in the analysis and discretized active spots (such as individual nanoparticles) effectively translate into a lower receptor coverage (and lower *Γ*_max_) over the full region. Concerning sensor geometry, it should be kept in mind that the optimal design of a single nanosensor element is a very different issue from the optimization of *how many* of those nanoobjects that should be used in the sensing application, *i.e.*, the total *A*. It is by all means a good idea to maximize the sensitivity of the individual sensing element, but one must then consider if it is really ideal to measure only on one such entity. The effect from geometry on mass flux is also discussed further in Section 2.2.

### 2.1. Small Samples

One straightforward reason for making a sensor small is that in the application under consideration *V* is so small that it becomes very difficult to expose the sensor to the sample solution without diluting it. Using the denotation in Figure 1, one can say that the sample volume *V* must be at least as large as the product *Ah* in order to establish proper contact. For instance, assume one wants to detect an analyte in one bacterial cell using a quartz crystal microbalance (QCM). We then have a sample volume below *V* = 10^−18^ m^3^, but an ordinary QCM is a very large sensor based on binding to a ∼1 cm^2^ sensor crystal [9]. This sensor test is clearly not feasible since the sample cannot be introduced properly (it requires an unrealistically small *h*). The situation then obviously requires diluting the sample by many orders of magnitude and *C* will become much lower, making efficient detection problematic (see further below). Instead, better performance will clearly be reached if *A* can be made smaller, at least when nothing else changes in the system (yet, it should be kept in mind that handling a volume as small as 1 fL will be problematic regardless of *A*). Given that miniaturization can be needed when *V* is extremely small, let us consider how common this situation is. Besides single cell analysis, one can also think of certain biopsy samples, in which case the sample volume would be on the order of *V* = 1 μL. (This is also a good measure of the smallest volume that can be easily handled with a common pipette.) In this case, one can actually expose a sensor as large as *A* = 1 cm^2^ using a *h* = 100 nm nanofluidic channel. Such a liquid cell would be quite extreme in terms of aspect ratio, but it is not necessarily an unrealistic configuration. However, it is probably preferable to have a sensor with slightly smaller *A*, suggestively on the order of 1 mm^2^.

Besides small *V*, the sample containing the analyte can in principle also be too “small” in terms of *number of molecules* available (*CV*). Even if *V* is reasonably large, there might not be many molecules present depending on the value of *C*. This is certainly the case for single cell analysis by QCM (in addition to the problem with too small *V*). Here the value of *A* naturally comes into play once more: Even if the sample liquid can be properly introduced to the sensor surface it is possible that *Γ*_max_ cannot be reached even if all molecules bind. Let us look at another example, namely the peptide hormone calcitonin, which is present at a mass concentration of *CM* ≈ 10 pg/mL in blood (the exact concentration indicates the state of medullary thyroid cancer). This is a very low concentration [10], making calcitonin one of the most challenging biomarkers to detect with surface sensitive techniques. The highest surface coverage possible will depend heavily on the receptor arrangement on the surface, but for relatively dense protein layers a value of *Γ*_max_ on the order of 100 ng/cm^2^ can be assumed [11]. Thus, for a large sensor with *A* = 1 cm^2^, a (standard) 10 mL blood sample will only provide calcitonin molecules enough to reach *Γ*/*Γ*_max_ = 0.1%. Does this mean a smaller sensor is needed, so that fewer molecules are needed to reach *Γ*_max_? The answer is actually no, not in practice, because it has implicitly been assumed that one *can* reach *Γ*_max_, but this holds true only for the very rare case of *irreversible* binding. Because of the low concentration of calcitonin and the limited affinity of recognition elements one can only expect a small fraction of the binding sites to be occupied even at equilibrium. Reversibility is usually illustrated with the common Langmuir isotherm for reaction limited binding [12]:

(1) $$\Gamma (t)=\Gamma_{\text{max}}\frac{k_{\text{on}}\;C}{k_{\text{on}}\;C+k_{\text{off}}}\;\left(1-e^{-(k_{\text{on}}\;C+k_{\text{off}})t}\right)$$

Here *k*_on_ and *k*_off_ are the rate constants for association and dissociation. The surface coverage at equilibrium (*t* → ∞) is *Γ*_eq_ and the fraction of binding sites occupied is given by:

(2) $$\frac{\Gamma_{\text{eq}}}{\Gamma_{\text{max}}}=\frac{k_{\text{on}}\;C}{k_{\text{on}}\;C+k_{\text{off}}}=\frac{1}{1+\frac{K_{D}}{C}}$$

Here *K*_D_ is the dissociation constant defined as the ratio of the association and dissociation reaction coefficients, *i.e.*, *K*_D_ = *k*_off_/*k*_on_. It is a measure of the strength of the interaction (low *K*_D_ represents high affinity). Preferably, *K*_D_ should be within the dynamic range of the sensor, *i.e.*, the range of analyte concentrations one wishes to detect, but it tends to be higher. Reaching *Γ*_eq_ = *Γ*_max_ requires *K*_D_ = 0, while in reality one can usually only get down to *K*_D_ ≈ 1 nM at best for antibodies [13]. Aptamers are no better in this respect [14]. Interestingly, it is also clear from Equation (2) that for nanosensors (e.g., *A* = 100 × 100 nm) and low *C* in general, one cannot expect that even a *single* receptor (on the order of ∼100 in total) has an analyte bound to it. Therefore, if one evaluates the sensor state at a given point in time, nanosensors will often actually give *Γ* = 0 instead of *Γ* = *Γ*_eq_ simply as a consequence of “molecular discretization” breaking the mathematically continuous model in Equation (2) [8]. One should also consider possible edge effects: do the receptors just at the edge of the active spot really behave the same way as the others? For smaller *A* the relative number of receptors just at the edge is higher.

Let us now look again at the calcitonin example when reversibility is accounted for. In this example *M* = 15 kg/mol, which gives *C* slightly below 1 pM and it is thus clear from Equation (2) that *Γ*_eq_ is around 0.1% of *Γ*_max_ for a high affinity receptor. As calculated above, this is exactly the highest possible *Γ* that the number of molecules in the sample allows us to reach when *A* = 1 cm^2^ and *V* = 10 mL. Thus, the advantage of sensor miniaturization is questionable: There seems to be enough molecules available even when *A* = 1 cm^2^. Strictly, this is not true since the Langmuir model assumes that the concentration in the sample is not reduced once some molecules have bound to the surface (molecules must be continuously supplied to the surface). Therefore a slightly smaller *A* is probably preferable, or more blood is needed, but pushing towards nanoscale sensors is obviously not necessary. Rather, it seems like a sensor on the microscale would represent more than enough miniaturization. I emphasize that this example was for what is arguably a “worst case scenario” biomarker and that there is much more molecular material available for other protein biomarkers in blood [10].

In order to establish equilibrium and approach reaction limited binding kinetics, it is often useful to implement *continuous flow* during the experiment. The volumetric flow will be approximately *Q* = *vhA*^1/2^ when the channel is as wide as the sensor (Figure 1). Further, one can let *τ* denote the time it takes to perform the sensor test. Naturally, the use of continuous flow then requires that neither *Q* nor *τ* reaches extreme values and for flow operation one clearly needs *Qτ* < *V*. This leads to a potential problem for larger sensors due to depletion of *V*, while smaller sensors should require a lower *Q* for the same flow velocity at the sensor (*v* determines how efficiently molecules are supplied to the sensor). Ignoring some extreme examples such as single cell analysis, do we have sufficiently large *V* in general also for operation under flow on large sensors? Let us once more look at the 10 mL blood sample together with a relatively large flow channel that has *h* = 1 mm and a long measurement with a duration of *τ* = 1 h. It is then clear that even for *A* = 1 cm^2^, one can implement flow at a rate of *v* ≈ 300 μm/s without depleting *V*. This is arguably a velocity that represents efficient delivery of molecules to the sensor [1]. How does the situation look like in other applications? For biosensors, the most common applications are related to medical diagnostics, food safety and environmental monitoring. In terms of medical diagnostics, one can normally acquire bodily fluids in amounts of *V* > 1 mL. It may be preferable to consume less volume, but *A* can decrease very many orders of magnitude before one reaches the nanoscale. Practically all samples from food industry or the environment are obviously even larger (people do not eat microscale portions and the environment is a very big place). This suggests that even for *A* on the mm scale, *V* is sufficiently large in most cases even for operation under continuous flow.

Looking further at the time of the sensor test, how can one know whether *τ* will not be so *long* that our sample is still too small for work under flow to be feasible? In other words, assuming the flow delivers new molecules to the sensor efficiently so that the system follows Equation (1) fairly well, is equilibrium reached? If not, a smaller sensor could be needed since it requires a lower *Q* to reach the same *v*. One clearly wants *τ* to be long enough for equilibrium establishment, so that *Γ* is close to *Γ*_eq_ and the signal maximized. At the same time, *τ* needs to be quick enough for the sensor test to be of practical use. It can be seen from Equation (1) that the system is close to equilibrium if *k*_off_*τ* *or k*_on_*Cτ* (not necessarily both) are comparable to one. Therefore, it is sufficient that *k*_off_^−1^ alone is comparable to *τ* regardless of *C* [8]. In other words, equilibrium can be reached as long as the molecular interaction lifetime is comparable to the time of the experiment. Although the desired *τ* will be quite different depending on the scenario, a typical high affinity *k*_off_^−1^ value is on the order of 10^5^ s [13] and it seems likely that one will often have *k*_off_^−1^ comparable to *τ*. Note that this does not mean that a high *k*_off_ is something desirable. A higher *k*_off_ will unfortunately always result in a lower *Γ*_eq_. It just happens to be the case that even the best receptors available normally provide interaction lifetimes comparable to the timescale of a typical experiment. It is also noteworthy that the commonly studied biotin-avidin interaction [15–17] is exceptionally strong with *k*_off_^−1^ > 10^7^ [18] and not at all representative for typical biomolecular interactions (although the *k*_on_ value is quite ordinary). One must be careful when using this interaction as a model system to draw generic conclusions about binding kinetics and sensor performance [17].

One can summarize this section by saying that in many real world sensor applications there will be sufficient volume and analyte molecules available, even if *A* is relatively large. However, the use of nanosensors is clearly motivated in some important cases when the number of analyte molecules available is very low, such as single cell analysis [19]. In some sensor applications, one aims to *study* a molecular interaction rather than detecting an analyte, such as in drug development and proteomics. In these cases, the researcher prepares the “sample” and can thus influence both *V* and *C*. The number of molecules available can then often be reasonably high without too much effort. For instance, drugs must obviously be possible to produce in relatively large quantities or else they would not be suitable candidates. Also, extensive research on protein production (see the Elsevier journal *Protein Expression and Purification*) has made it possible to produce quite large amounts of many proteins of interest, at least those that are water soluble.

### 2.2. Increased Flux

The effect from reduced *A* on the incident diffusive flux of molecules is perhaps the least discussed yet the most obvious advantage of miniaturized sensors, although it only applies for binding that is strongly influenced by mass transport [20]. For fully *diffusion limited* binding to a planar infinite surface in contact with a stagnant sample, *Γ* is a function only of concentration and the diffusion constant *D* of the analyte [12]:

(3) $$\Gamma (t)=2\mathrm{CM}\sqrt{\frac{\mathrm{Dt}}{\pi }}$$

Equation (3) represents a sensor that captures molecules immediately when they are at the surface (*k*_on_ → ∞), which never saturates (infinite *Γ*_max_) and the binding is implicitly considered to be irreversible (*k*_off_ = 0). Naturally, this can only ever be valid for the initial binding phase well before equilibrium is reached [12]. Even though reaction kinetics are effectively ignored in Equation (3), it is possible to say something about the upper performance limit of the sensor only from knowing *D*. This is because a molecule can diffuse to the surface without binding to it, but not bind to the surface without first diffusing to it. Looking again at proteins as model analytes, one has *D* around 10^−10^ m^2^/s [21] and Equation (3) predicts that reaching sufficiently high *Γ* for detection, typically *Γ* = 0.1 ng/cm^2^ [1,22], may unfortunately take years if *C* is below 1 pM (e.g., calcitonin in blood).

**Figure 2.**
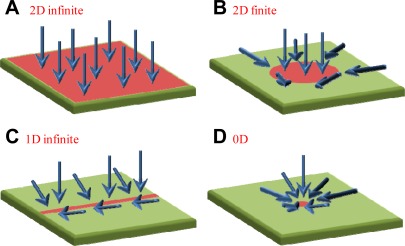
Influence from sensor size on incident flux in a system with diffusion limited binding from stagnant liquids. The arrows show the directions of the analyte flux, assuming that nothing binds outside the active sensor region. The planar infinite surface in (**A**) gives the lowest average incident flux. If the spot is smaller, as in (**B**), the binding rate will become higher, especially at the edges. The binding rate is also higher in (**C**), showing a one dimensional sensor, which gives an incident flux with cylindrical symmetry. The highest average flux to the active area occurs for radial symmetry and a point-like sensor, as shown in (**D**).

Although only approximate analytical solutions are available for diffusive flux to other sensor geometries, it is clear that the infinite surface geometry provides the lowest flux of molecules to the sensor [23]. Therefore, smaller sensor spots will tend to cause an increased flux into *A* due to the edges [20]. The increased flux associated with smaller sensors is conceptually illustrated in Figure 2 for different architectures. When diffusion determines binding rate, the smaller sensors will result in higher ∂*Γ*/∂*t* because their geometry induces increased flux at the borders. One can also think of this phenomenon as an effect of sensor dimensionality: Equation (3) represents the slowest type of binding because the depletion zone only grows in one dimension, but “wires” or “dots” have depletion zones that grow in two or three dimensions [23]. For the one dimensional problem [Equation (3)], the depletion zone at the sensor has a characteristic thickness determined by the independent Brownian motion of the molecules:

(4) $$\bar{z}=\sqrt{2\mathrm{Dt}}$$

It follows that the ideal sensor in this context is infinitely small and preferably also somehow suspended from the supporting surface and fully introduced into the sample. Such a sensor can “suck in” analyte molecules from all directions to a single point, thereby maximizing the flux. Notably, heat transport follows the same principles and if heating is an issue for the sensor under consideration, miniaturization can lead to more efficient cooling.

As illustrated in Figure 2, a smaller sensor may result in a higher *Γ*, but one must then consider in what situations this conclusion applies. It is quite rare to have purely diffusion limited binding from a stagnant liquid. Indeed, the most obvious way of countering the constraints imposed from diffusion, namely by introducing flow [1], has so far been ignored in this analysis. As discussed in the previous section, many samples of relevance are large enough for measurements under constant flow. Miniaturized sensors actually make it more difficult to enhance the binding through convection. This is simply because for small *A*, molecules tend to pass by the sensor element without binding to it [7,8]. To understand this conceptually, consider once more our flow channel geometry in Figure 1. For flow to be reasonably efficient in increasing the incident flux to *A*, each molecule must spend a time above the sensor equal to the time it takes to diffuse down to the surface on average [8]. (The dimensionless Peclet number, which represents the ratio of these times, should be one.) The corresponding flow, denoted *Q*_capt_, can be calculated by using Equation 4 and integrating across the channel. Since the average time a molecule spends above the sensor is *Ah*/*Q* one gets [1]:

(5) $$Q_{\text{capt}}=\frac{\mathrm{Ah}}{\frac{1}{h}\;\int_{0}^{h}\frac{z^{2}}{2D}\;dz}=\frac{6\mathrm{AD}}{h}$$

**Figure 3.**
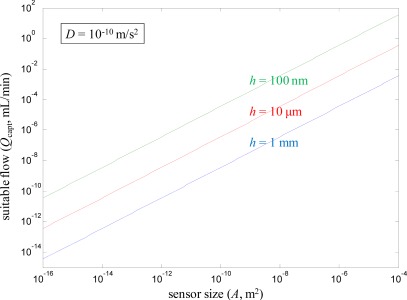
Suitable volumetric flow for efficient delivery of molecules to the sensor as a function of sensor size. The geometry is based on the channel in Figure 1 and the flow is calculated from Equation (5). A diffusion constant of 10^−10^ m^2^/s is assumed for the Brownian motion perpendicular to the surface. Flow rates higher than *Q*_capt_ will still increase the delivery rate of molecules but relatively little.

The flow rate calculated from Equation (5) represents a compromise between enhancing mass transport to the sensor while preserving decent capture efficiency, *i.e.*, the probability that a molecule introduced with the flow actually encounters the sensor surface should be around 50%. Of course, if one has practically unlimited amounts of sample volume available, it is still possible to increase the flow (*Q* > *Q*_capt_) and further enhance ∂*Γ*/∂*t*. However, the effect becomes much weaker and *Γ* approaches a *Q*^1/3^ dependence [8], which is also what happens for large channels [24] (*h* → ∞). Therefore, Equation (5) is still important because it indicates the absolute flow rate at which it becomes difficult to achieve further enhancement from flow in diffusion limited systems. Figure 3 shows results from using Equation (5) to calculate *Q*_capt_ for different *A* and a few different *h*, fixing *D* as 10^−10^ m^2^/s. Clearly, a small *A* is associated with a low *Q*_capt_, although the flow channel height also is important. The larger sensors (up to 1 cm^2^) have no problems capturing molecules even at relatively high *Q*. In contrast, the nanosensors (*A* down to 10 × 10 nm) essentially cannot be used together with flow at all if good capture efficiency should be maintained, regardless of the flow channel height. Admittedly, for small sensors some enhancement is still possible even in the *Q*^1/3^ limit since *Q* can be increased so many orders of magnitude above *Q*_capt_. Note that it has been assumed that the flow channel width is equal to the width of the sensor spot, which is an arbitrary rectangle. It should also be kept in mind that for binding controlled by diffusion under convection one gets an inhomogeneous *Γ* in the direction of the flow [24].

In summary, one can say that although sensor miniaturization may help to increase the binding rate from stagnant liquids in the diffusion limited case, it also tends to spoil the enhancement which is possible by introducing flow. When the sensor is larger, it is possible to introduce higher flow rates and still expect the sensor to capture a large fraction of the molecules. A complete optimization requires taking the sensor and flow channel geometry into account, but one must also consider how much sample there is available and to what extent diffusion controls the binding rate. Interestingly, *Q*_capt_ is proportional to the number of molecules introduced to the sensor but also proportional to *A* [Equation (5)]. Therefore, given that Equation 5 well represents the highest flow at which a reasonably high fraction of the introduced molecules bind (for any given *A*), *Γ* becomes essentially *independent* of sensor size in diffusion controlled systems (*Γ* equals the number of molecules captured divided by *A*). This suggests that focus should be put on minimizing *h* instead of *A*, *i.e.*, one should work towards efficient flow configurations [16,25]. It should then be kept in mind that the high pressure drops associated with narrow microfluidic channels may be problematic.

### 2.3. Multiplexing

Many surface sensitive techniques are compatible with detection in arrays, even with parallel readout. Examples include SPR imaging [26], piezoelectric resonator arrays [27] and cantilevers [3]. It is obvious that a higher degree of multiplexing can be reached if each sensing element is smaller. So how small should *A* (the size of one sensor element) preferably be in order to reach the degree of multiplexing necessary? Although this will clearly vary from case to case, it seems hard to suggest the use of nanosensors just for the sake of multiplexing. In many biosensor applications, such as detection of biomarkers in blood or pathogens in food, it seems like one would want to detect around 100 analytes at the most, which will not require very small sensing spots. But one can anyway look at an extreme example: assume one wants to scan the entire human *proteome*, including alternate splicing and posttranslational modifications, on a 1 cm^2^ chip. This corresponds to almost ∼10^6^ analytes, but one only needs sensor spots of about 10 × 10 μm. The only practical example where so many interactions need to be measured is most likely antibody library scans [28]. It thus seems rarely necessary to approach the nanoscale just for the sake of sufficient multiplexing unless the total size of the sensor chip must be very small for some reason. Another reason why it is questionable to have small *A* just for the sake of multiplexing is that depending on the transducer mechanism there are various practical limits on parallel readout of many individual close packed sensor spots. Also, even if readout is possible, handling data from millions of channels operating in real-time is clearly problematic.

In addition to the abovementioned one must also consider the limitations of serial surface modification methods [28]. Multiplexed detection is based on the possibility to functionalize one sensor spot with one type of receptor only, with the affinity for its analyte preserved. For the case of protein receptors, the most efficient patterning strategies operate with microscale resolution [29]. For instance, the fastest and most reliable method for array functionalization is arguably still microdispensing, which has a spatial resolution limited to ∼100 μm for the liquid droplets on the surface (depending on contact angle). Scanning probes techniques [30] can introduce chemical patterns on very small regions but operate slowly and are not compatible with fragile biological molecules. Electrochemical methods [31] and material specific chemistry [17] can be used to pattern nanostructures, but again only in a slow serial manner. It seems clear that with the toolset available, functionalization of a single nanosensor element is feasible, so the functionalization issue is not a valid criticism against nanosensors. However, it is still fair to claim that efficient functionalization of nanometric sensing *arrays* is highly problematic, which in turn questions the value of miniaturization just for the sake of multiplexing.

### 2.4. Detection of Single Binding Events

**Figure 4.**
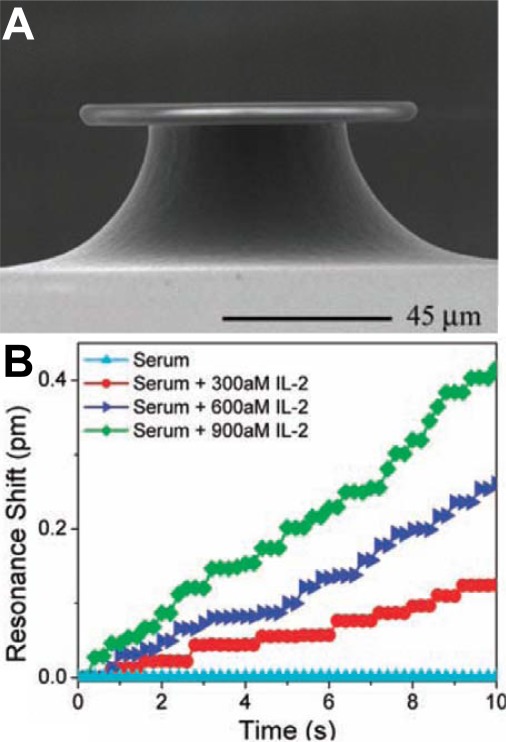
First report of label-free biomolecule detection from serum with single molecule resolution using an optical microcavity (whispering gallery mode) resonator. The resonator shape and size is illustrated in (**A**). Examples of binding curves with discretized signal steps is shown in (**B**) when interleukin binds to the sensor surface. Reproduced with permission from Reference [35] (Copyright 2007 American Association for the Advancement of Science).

Resolving single molecule binding events appears to be a dream for most researchers working with sensor development. When the sensor signal is determined by *Γ*, reducing *A* is the most obvious way to get a fewer number of molecules corresponding to the same signal. The smallest sensors to date are probably single plasmonic nanoparticles [32], which seem to be just at the limit of resolving single biomolecular binding events [33,34]. It is clear that as long as *A* is ∼1 μm^2^ or higher, single molecule resolution cannot be realized because the detection limit in *Γ* is always so high that the signal to noise ratio is lower than the number of bound molecules. It thus appears reasonable to claim that nanosensors are required for resolving single molecules. In this context it is interesting to note that detection of single molecule binding events were first reported with a relatively large (microscale) optical sensor (Figure 4) utilizing so called whispering gallery modes [35]. Since then the heating mechanism suggested to be responsible for the large signal per molecule has been questioned [36], but development of the sensor continues [6]. Although it represents an impressive engineering achievement worthy of attention, it is worth to ask the question what the single molecule resolution feature contributes with in terms of sensor efficiency.

Unfortunately the literature seems to confuse single molecule *resolution* with single molecule *detection*, which is an extreme case of low *CV*. The single molecule detection problem has already been discussed above and this section deals with the concept of single molecule resolution, which is a prerequisite for (but does not directly enable) the possibility to detect a single molecule. This is because even if single molecule binding events can be resolved, it is still very problematic to guide the single molecule to the (extremely small) active sensor site. Looking at the sensor in Figure 4(A) as an example, it has not been proven that *every* bound molecule gives rise to a step higher than the noise in the binding curve [Figure 4(B)]. Indeed, in surface sensitive techniques, especially those compatible with extreme miniaturization, the same molecule normally gives a different signal depending on where on *A* it binds [17]. In other words, variations in the *magnitude* of the step-like signal from a molecule will not represent useable information because the sensitivity is not homogenously distributed to start with. In addition, surface sensitive techniques rarely make it possible to say anything about which receptor that was responsible for an observed binding event [37]. The question is then what new information one can obtain from monitoring the *number* of discretized binding/unbinding events during a certain time.

The general argument why studying single molecules (or nanoparticles, quantum dots *etc.*) is important is that it enables the observation of different molecular states or interaction mechanisms. Such effects are averaged out and difficult to observe in ensemble measurements. However, it is important to distinguish between scientific insights on molecular behavior and the efficiency of surface-based sensors. It is only when one uses sensors to investigate the nature of a molecular interaction that single molecule resolution may be relevant. Even then it is relatively hard to find concrete examples where it is obvious that single molecules must be resolved. One special case is the study of conformational changes during a molecular interaction, which may be interesting for biomacromolecules [38,39]. Another exception is the case when two molecules bind to each other in different ways, leading to different *k*_on_ and *k*_off_ values [Equation (1)]. For instance, an antigen can have two epitopes that an antibody can recognize. By studying individual binding events, it becomes relatively easy to see that two types of interactions are occurring [37]. On the other hand, these effects are to some extent observable also in ensemble measurements (*i.e.*, larger sensors) by proper analysis of binding/unbinding kinetics [40]. For instance, to make Equation (1) valid, efficient flow is needed during the association to avoid depletion and during the dissociation phase to avoid rebinding. If the curves acquired under such circumstances clearly differ from single exponentials, it shows that the interaction is more complex than simple first order kinetics and other models can be tested instead. Yet, single molecule resolution will naturally help illuminate the mechanism. This is especially so if it is due to heterogeneity in the population of molecules.

Many people consider single molecule resolution to be the obvious ultimate limit of sensor performance [32–35] and miniaturization is clearly needed if one should attempt to reach this goal. I suggest that this dogma should be reconsidered. By pushing towards single molecule resolution one has implicitly assumed that the detection limit in terms of number of bound molecules (*ΓA*) is more important than the detection limit in terms of *Γ*. As I have tried to explain, in many real world sensor applications there are plenty of molecules available and it is better to think in terms of concentration. Given that you have obtained single molecule resolution, I suggest asking questions like: What new information have I gained by observing single molecules? In which real-world application will this sensor be more efficient than its larger counterpart? What would my detection limit in *Γ* be if I had made my sensor larger? If you do get close to single molecule resolution, you will most likely be looking at relatively large analytes [34] or lack real-time data [33]. Indeed, you will likely have a poor detection limit in terms of surface coverage because of the problems associated with miniaturization, which will be considered next.

## 3. Problems with Miniaturization

Section 2 looked at possible advantages with sensor miniaturization and I have given arguments suggesting that even though there are benefits from smaller *A*, in many cases they are overrated. But even if there is not so much to gain from miniaturization, it is still fair to ask why one should *not* miniaturize? Is the worst case scenario that the performance gain from miniaturization is smaller than expected? Indeed, if there are no particular disadvantages with making sensors smaller, one might as well do it for what it is worth. There are, however, usually several problems with miniaturization. I will try to give a brief overview of such issues here although the dominating type of problem naturally varies much depending on the sensor type. There are a few obvious points to start with. For one thing, it is usually much more complicated to fabricate a nanosensor compared to a larger one, especially for mechanical [3,27] and electrical [4] transducers. More advanced and expensive equipment is usually needed for nanoscale precision, especially for microelectromechanical systems. Nanosensors are also naturally more complicated to handle and they can be challenging to observe even in microscopes. One important exception is plasmonic nanoparticle sensors which are actually relatively simple to produce and measure on [32]. Still, the most important concern with miniaturized sensors is probably the decrease in measurement precision, which results in a poor detection limit. One can distinguish between short-term *noise* issues (typical timescale up to 1 s) and long term *stability* issues (timescale of minutes or more). Noise can often be well described by statistical laws, while stability issues such as baseline drifts are more complicated since they can be caused by so many factors. I will briefly describe how miniaturization can influence noise and stability. Unfortunately there are few studies on this topic, which is another fact I wish to highlight.

Regarding noise, it should first be noted that essentially all optical and electrical surface sensitive techniques operate by monitoring a flux of photons (intensity) or electrons (current) and detecting changes in this flux in response to molecular binding to the surface. Many optical sensors operate by measuring several intensity values using photodetector arrays corresponding to different wavelengths or angles. Still, the principle remains the same and each photodetector operates independently [15]. In addition, electrical or optical readout is normally used [41], even if the transducer is mechanical in nature [5] (as an example, consider how cantilever deflection is measured). Furthermore, any photodetector essentially converts the photon flux into a current, so it seems fair to say that the sensor output consists of a current for simplicity. A part of this current consists of “real” electrons, *i.e.*, those associated with what is happening at the sensor surface, which can be denoted *I*. However, the sensor output will also contain false electrons due to intrinsic noise in the instrumentation, generating a current that can be called *I*_noise_. Molecular binding to the sensor causes a change in *I* which is, in the majority of cases, proportional to the initial value of *I*. In an optical sensor, the probing light intensity or the initial current that is measured will change with a certain percentage in the experiment. (For instance, the refractometric sensitivity of a plasmonic nanostructure has a certain value which is obviously independent of how strong lamp one uses to measure the spectrum.) Thus, one can say that the current changes from *I* to *αI*, where *α* is simply a number close to unity with an exact value that depends on the given change in *Γ*.

It should now be noted that both *I* and *I*_noise_ are in fact random variables fluctuating around an average value and this is what causes noise in the measurement. Any flux of discrete independent entities (electrons or photons) follows Poisson statistics, *i.e.*, shot noise, which means that the standard deviation of *I* is *I*^1/2^ [15]. It is then possible to express the signal to noise ratio of the measured current:

(6) $$\frac{\text{signal}}{\text{noise}}=\frac{\alpha I-I_{\text{noise}}-\left(I-I_{\text{noise}}\right)}{\sqrt{{\left(\sqrt{I}\right)}^{2}+\text{var}\left(I_{\text{noise}}\right)}}=\frac{I(\alpha -1)}{\sqrt{I+\text{var}\left(I_{\text{noise}}\right)}}$$

Here var(*I*_noise_) represents the variance of the “false” part of the measured flux (this variance is unknown but likely increases with the average *I*_noise_). It can be seen from Equation (6) that signal to noise increases with *I* and that one would like to have *I* >> *I*_noise_. Intuitively, this is expected since one wants as many as possible of the measured electrons to be associated with what is happening on the sensor surface and not an artifact of the detector. So what does all this have to do with miniaturization? The answer is that it is obviously harder to get high *I* in the case of a small sensor. No other parameter in Equation (6) has anything to do with *A* (not even α as long as the sensor operates by the same principles regardless of *A*). In an electrical sensor, one will clearly have higher *I* for higher *A*, often in a linear relation. The situation is the same for the case of optical readout because one will have a higher photon flux to analyze if a larger area is illuminated. However, it is possible to focus the probing light and in some cases the same noise level can be reached for a smaller *A*, although issues with heating may come into play. There are even some situations when noise can increase with *A*. This can be the case in electrical measurements where the dominating noise is due to the working electrode acting as an antenna (this can be considered a part of *I*_noise_ although it is not exactly from the instrument itself). It should be noted that in the purely shot-noise dominated case (*I* >> *I*_noise_) the signal to noise ratio *continues* to increase with *I* in a square-root dependence. In summary, through Equation (6) it is possible to understand why miniaturization often leads to higher noise in the measurement.

**Figure 5.**
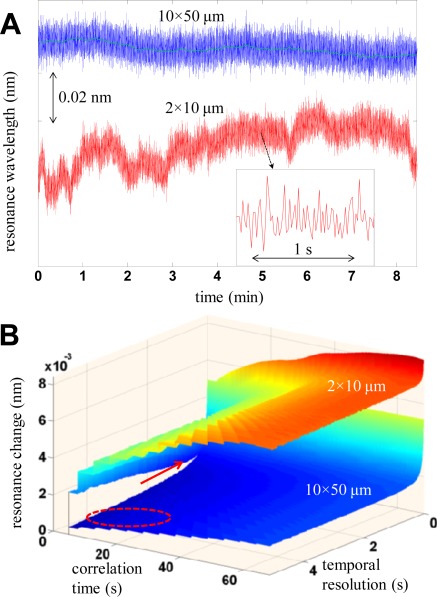
Example of stability differences when performing microspectroscopy on areas of different size (10 × 50 or 2 × 10 μm). The sensor output parameter is the resonance wavelength of plasmonic gold nanodisks on the surface (in nm). The baseline of the idle system is monitored in (**A**) showing the same short-term noise for both *A*, but improved stability for the higher *A*. The change in sensor parameter between two acquisitions as a function of the temporal resolution and the elapsed time between the acquisitions is shown in (**B**). The arrow and circle indicate the lowest noise for the smaller and larger A respectively. Reproduced with permission from Reference [15] (Copyright 2009 American Chemical Society).

When it comes to stability issues, there are some studies performed on optical sensors showing that mechanical stability often becomes a problem for small sensors. For instance, it has been shown that the resolution in single nanoparticle spectroscopy suffers heavily from small changes in position of the particle with respect to the surrounding optical components [42]. Another example is given in Figure 5(A), which shows an example from transmission mode spectroscopy on the microscale. Two differently sized areas are probed and the sensor output parameter (here resonance wavelength) is monitored when the system is idle. For *A* = 10 × 50 μm, the baseline is relatively stable while for *A* = 2 × 10 μm there are clear stability problems and additional fluctuations occur on a timescale of ∼1 min even though the noise level is the same (on the short timescale). This is further illustrated in Figure 5(B), which shows the differential of two sensor readouts as a function of number of averages and time elapsed between the readouts. Although the same noise is observed for both values of *A* at the highest temporal resolution, a lower noise level is possible for the higher *A* though averaging while the stability problems associated with the smaller *A* hinders noise reduction. The improved stability for larger *A* was attributed to small movements of the surface in relation to the optical setup [15]. Over very short time periods, the same noise was observed for both *A* because in this particular case *I* was limited by the detector dynamic range. Of course, this single example does not say much but about the general stability of nanosensors compared to their larger counterparts. Most likely, one can achieve good stability also for miniaturized sensors, but it seems fair to say that smaller sensors will tend to become less robust. They will likely require a more controlled environment and complicated experimental setup.

## 4. Conclusions

I have presented an overview of the advantages and problems associated with making smaller sensors. The sensor is assumed to operate through some sort of surface sensitive mechanism, *i.e.*, label-free surface-based transduction. Other than that, the conclusions are essentially generic regardless of the transducer principle. Focus has been put on detection in liquids and biosensor applications. The most common reasons for miniaturizing have been presented and it has been shown that they are often overrated, *i.e.*, nanosensors do not necessarily provide any better performance. Instead, the sensors become more complicated and performance in terms of detection limit is often worse due to high noise and poor stability. However, this depends on how the limit of detection is defined. In particular, one must distinguish between detection limits in terms of surface coverage and number of molecules. In most cases surface coverage is what matters and it is for good reasons that detection limits in commercial systems are specified in terms of surface coverage. Still, in some cases the number of molecules available is so low that it actually becomes the relevant quantity and sensor miniaturization is necessary.

Even though the guidelines presented in this review are meant to be generic for all sensor types, it is difficult to draw any definite conclusions about the optimal size of a sensor in absolute numbers. Such an absolute number will depend on the transducer mechanism and the application, but all in all it seems fair to say that the microscale represents a good compromise between utilizing any positive effect from miniaturization while avoiding its complications. As a rule of thumb, if *A* is around 100 × 100 μm it should be possible to generate a high incident flux of molecules by continuous flow and perform multiplexed detection in arrays. At the same time, the signals from the active sensor will probably be high enough to avoid instrumental noise and mechanical stability is likely less of a problem. Although this is a very rough guideline, it seems clear that in most cases the optimal sensor size is at least well above that in sensors based on single nanoscale objects. Again, this does not exclude that nanosensors will be important in certain specific applications. In particular, the situation may be very different if one considers *in vivo* sensors or implants in the body. It should also be noted that this review does not at all represent criticism against nanotechnology in general since many transduction mechanisms originate from the properties of nanostructures. However, the optimization of an individual such sensor is a different matter. I only wish to emphasize that even if a single nanostructured object *can* operate as a sensor, it is often preferable to measure on an ensemble of such nanoobjects.
